# Knockdown of long non-coding RNA CCAT2 suppressed proliferation and migration of glioma cells

**DOI:** 10.18632/oncotarget.13242

**Published:** 2016-11-09

**Authors:** Hua Guo, Guowen Hu, Qing Yang, Pei Zhang, Wei Kuang, Xingen Zhu, Lei Wu

**Affiliations:** ^1^ Department of Neurosurgery, The Second Affiliated Hospital of Nanchang University, Nanchang 330006, China; ^2^ Department of Respiratory Medicine, The Second Affiliated Hospital of Nanchang University, Nanchang 330006, China

**Keywords:** qRT-PCR, quantitative real-time PCR, lncRNA, long non-coding RNA (lncRNAs), CCK-8, cell counting kit-8

## Abstract

Long non-coding RNA colon cancer-associated transcript 2 (CCAT2) is commonly investigated in a number of cancers. However, little is known of its expression and biological function in glioma biology. In the current study, we used quantitative real-time PCR (qRT-PCR) to determine the expression of CCAT2 in glioma tissues. We found that expression of CCAT2 was up-regulated in glioma tissues and significantly correlated with the advanced tumor stage (III/IV). Functional assays *in vitro* and *in vivo* demonstrated that knockdown of CCAT2 could inhibit proliferation, cell cycle progression and migration of glioma cells. Further analysis indicated the effect of CCAT2 knockdown on glioma cell phenotype through inhibiting Wnt/β-catenin signal pathway activity. Thus, our study provides evidence that CCAT2 may function as a potential biomarker for glioma.

## INTRODUCTION

Glioma is the most commonly diagnosed malignancy of central nervous system [1], leading to significant mortality worldwide annually. Emerging evidence suggests that numerous genetic and epigenetic alterations involved in glioma progression. Recently, most studies have focused on a newly discovered class of noncoding RNA, long non coding RNAs (lncRNAs), which served as major player in gene expression and the regulation of crucial biological roles in cellular physiology [2–4]. And it is well recognized that some altered expression of lncRNAs has been frequently linked with cancer pathogenesis [5–8], providing new insight into the genetic and molecular mechanisms of the cancer.

Colon cancer-associated transcript 2 (CCAT2), a novel noncoding RNA mapping to the 8q24 gene desert region, was firstly identified as oncogenic lncRNA in microsatellite-stable colorectal cancer. Increasing evidence shows that CCAT2 was shown to be consistently upregulated in esophageal squamous cell carcinoma, gastric cancer and breast cancer [9–11]. In addition, Ling et al found that CCAT2 is aberrantly expressed in colon cancer and the upregulation of CCAT2 is involved in promoting the growth and metastatic phenotype of colon cancer cells [12]. The biological function of CCAT2 as an oncogene in various human cancers suggested that it might be a potential and improved biomarker in the therapeutic of patients. However, the expression and detailed function of CCAT2 in glioma remains largely unknown and needs to be investigated.

In this study, we explored the expression pattern of CCAT2 in glioma patients and its correlation with clinicopathological factors of glioma. Furthermore, the biological function of lncRNA-CCAT2 in glioma cell' proliferation, cell cycle, and migration was examined *in vitro* and tumorigenicity in the nude mouse model was also investigated.

## RESULTS

### Expression of CCAT2 in glioma tissue samples

**Figure 1:**
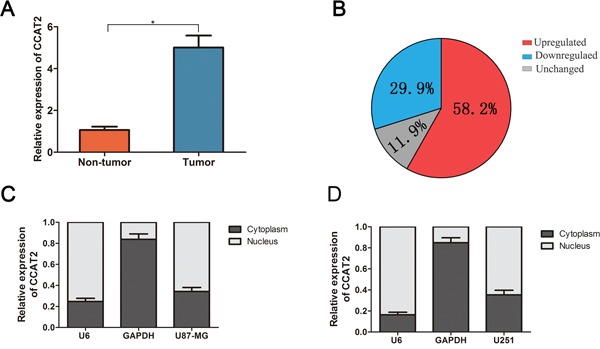
Quantitative determination of CCAT2 by qRT-PCR in glioma tissues and glioma cell lines **A.** Relative expression level of CCAT2 expression in glioma tissues and adjacent non-tumor tissues was measured by qRT-PCR (N=134). **B.** The percentage of glioma patients with lncRNA-ATB expression level (unregulated, downregualted and unchanged). RNA relative expression levels were normalized against the gene *GAPDH* transcript expression levels. ^*^P=0.023, paired *t*-test. The nucleus localization of CCAT2, nucleus-retained *U6* and cytoplasmic control transcript *GAPDH* in U87-MG cells **C.** and U251 cells **D.**

To investigate the potential biological functions of CCAT2 in glioma, we evaluated the CCAT2 mRNA expression by qRT-PCR in paired glioma tissues and adjacent normal tissues obtained from 134 patients with glioma. The expression of CCAT2 was significantly higher in glioma tissues than in adjacent normal tissues (Figure 1A). And 58.2% (78 out of 134) glioma tissue samples showed high expression of CCAT2 mRNA compared with that adjacent normal tissues, while 11.9% (16 out of 134) tissues showed no change (Figure 1B). Further, levels of CCAT2 in nuclear and cytoplasmic fractionated U87-MG and U251 cells revealed that CCAT2 was mainly existed in the nucleus of glioma cells (more than 65%) (Figure 1C and 1D). Clinicpathological characteristics of the 134 glioma patients presented in Table 1 showed that high expression of CCAT2 was significantly correlated with higher WHO grades (III/IV). In addition, to strengthen tissue expression analysis, we enrolled another 56 paired glioma tissues and adjacent normal tissues to confirm the expression level of CCAT2. Consistent with the above results, qRT-PCR analysis showed that expression level of CCAT2 in 56 glioma tissues was higher compared with matched noncancerous tissues, and patients with advanced TNM stage was correlated with increased CCAT2 expression (*P*<0.05; Supplementary Figure 1A and 1B).

**Table 1:** Association between lncRNA CCAT2 expression and clinicopathological features in glioma

Variables	N of cases	Relative CCAT2 expression				*P*_value_
		High		Low		
**Age** (median; range)	55 (11-81)					
<55	56	35	(62.50)	21	(37.50)	0.318
≥55	78	42	(53.85)	36	(46.15)	
**Gender**						
male	94	53	(56.38)	41	(43.61)	0.698
female	40	24	(60.00)	16	(40.00)	
**Tumor size (cm)**						
<5	85	45	(52.94)	40	(47.06)	0.163
≥5	49	32	(65.31)	17	(34.69)	
**Tumor Grade**						
I/II	58	26	(44.83)	32	(55.17)	**0.011**
III/IV	76	51	(67.11)	25	(32.89)	

### CCAT2 knockdown suppressed the proliferation, cell cycle progression and migration of glioma cell lines

**Figure 2:**
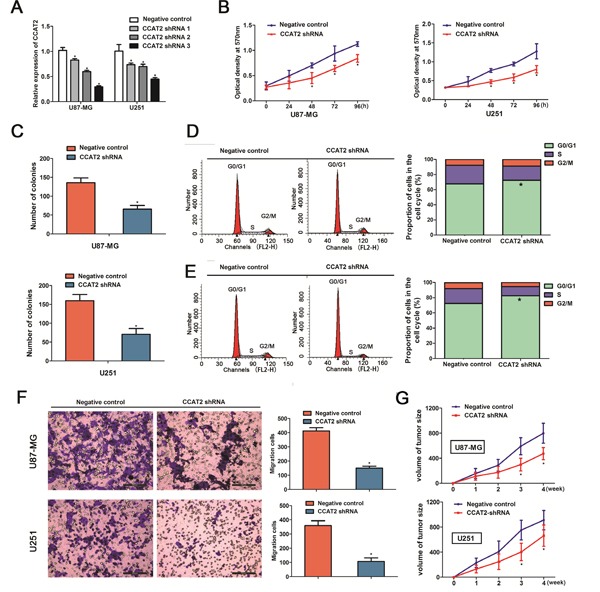
Knockdown of CCAT2 in glioma cell lines suppressed cell proliferation, cell cycle progression and migration of glioma cells **A.** The Knockdown effects of CCAT2 were measured by qRT-PCR in glioma cells transfected with shRNAs or its negative controls. Representative results for cell proliferation rate were evaluated by using CCK-8 **B.** and colony formation **C.** assays at the indicated days. The two-sided Student's *t*-test was used to calculate *P* value. Representative micrographs (left) and quantification (right) of U87-MG cells **D.** and U251 cells **E.** transfected with shRNA or its negative control by flow cytometric analysis with PI staining. **F.** Cell migration was inhibited by the effect of CCAT2 knockdown in the indicated glioma cells. Results are represent as mean±SD (Two-sided Student's *t*-test; n=3, *P*=0.011 for U87-MG cells and *P*=0.014 for U251 cells, compared with controls, respectively). **G.** Tumor volumes were measured in CCAT2-shRNA groups and negative control groups. Downregulated of CCAT2 significantly inhibited tumor growth *in vivo*. Results are represent as mean±SD (Two-sided Student's *t*-test was used; n=6, *P*=0.010 for U87-MG cells and *P*=0.018 for U251 cells, compared with controls at the fourth week).

The above results prompted us to evaluate the biological role of CCAT2 in glioma cells. U87-MG and U251 cells were seeded in 6-well plates, and the specific lentiviral vector expressing CCAT2 shRNAs was transected to glioma cell lines to determine its effect on the proliferation of glioma cells *in vitro*. Figure 2A showed that CCAT2 was efficiently silenced using shRNAs (shRNA1, shRNA2 and shRNA3) in U87-MG and U251 cells compared with its negative controls. The CCAT2 shRNA3 against the CCAT2 mRNA was choose to use in the following experiments. Using CCK-8 and colony formation assays, we found that glioma cells in CASC2 shRNA groups grew significantly slower compared with the cells in the negative control groups on day 4 (Figure 2B). Accordingly, consistent with the proliferation assay, results from colony formation assay revealed that silencing CCAT2 expression significantly inhibited the colony formation of U87-MG cells (Figure 2C), similar results were also observed in U251 cells.

Having found the effect of CCAT2 downregulation on the proliferation of glioma cell lines, we then examined the impact of decreased expression of CCAT2 on cell cycle in glioma cells. Flow cytometric analysis showed a decrease in the percentage of cells in the S phase and a marked accumulation in the percentage of cells in the G0/G1 phase in the CCAT2 shRNA groups in U87-MG cells (72.48±1.32% vs. 67.59±1.07 in G0/G1 phase; 18.66±1.12% vs. 24.65±1.35%, in S phase, *P*<0.05, Figure 2D), compared with the respective control groups. Flow cytometric analysis of U251 cells showed the same tendency as the U87-MG cells (82.57±1.27% vs. 72.66±1.21 in G0/G1 phase; 12.14±1.52% vs. 19.45±1.43%, in S phase, *P*<0.05, Figure 2E). Furthermore, transwell assays indicated that the down-regulation of CCAT2 in the glioma cells resulted in a marked decrease in cell migration (Figure 2F).

### Knockdown of CCAT2 reduced tumor growth *in vivo*

Given that decreased expression of CCAT2 inhibited proliferation *in vitro*, we validated the *in vitro* effects of CCAT2 downregulation *in vivo* by injecting the indicated stable expression glioma cell lines with CCAT2 shRNA or its respective controls into the brains of nude mice. As shown in Figure 2G, down-regulation of CCAT2 expression had significantly reduced tumor growth compared with that of respective groups.

### Downregulation of CCAT2 decreased the downstream genes expression of Wnt/β-catenin signaling pathway

**Figure 3:**
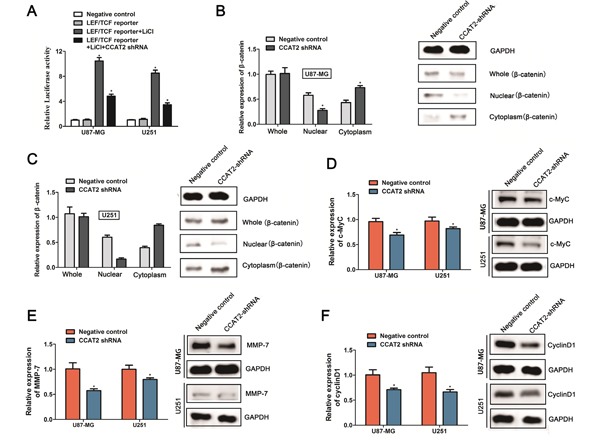
Knockdown of CCAT2 inhibits the downstream genes expression of Wnt/β-catenin signaling pathway **A.** CCAT2 knockdown significantly suppressed the LEF/TCF promoter activities in glioma cells treated with 10mM LiCl. Statistical data were presented as mean ± SD (n=3) from three independent experiments. *P<0.05, Two-sided Student's *t*-test. The β-catenin mRNA (left) and protein (right) levels quantified in whole cell extracts or different cellular compartments from U87-MG cells **B.** and U251 cells **C.** expressing CCAT2-shRNA or scramble shRNA (negative control). Effects of CCAT2 knockdown by shRNA on the mRNA level and protein expression of c-Myc **D.**, MMP-7 **E.** and CyclinD **F.** in glioma cell lines (U87-MG and U251). MRNA levels and protein levels were measured by qRT-PCR analysis and western blot analysis, with *GAPDH* used as a control. Data are presented as the mean ± SD (n=3). *P<0.05, Two-sided Student's *t*-test.

It has been well established that the cellular localization of β-catenin, which is a key component in the Wnt/β-catenin signaling pathway could affect downstream genes expression, and play crucial roles in normal development and carcinogenesis. Zhang et al revealed that down-regulated CCAT2 expression combined with Wnt signaling inhibitor FH 535 which affecting the nuclear accumulation of β-catenin thus attenuated Wnt/β-catenin signaling transcriptional activity in breast cancer. To further explore the specific mechanism regulated by CCAT2 in the proliferation of glioma cells, we tested the effect of CCAT2 on Wnt/β-catenin signaling using a reporter LEF/TCF promoter dual-luciferase construct. After 24 hours of transfection with LEF/TCF reporter, Glioma cells treated with extracellular stimuli factor LiCl (10mM), which activates Wnt signaling activity, indeed led to a notable >8-fold increase in the luciferase activities of the reporter (Figure 3A). In addition, repression of CCAT2 treated with CCAT2-shRNA markedly inhibited the reporter activity of LEF/TCF induced by LiCl compared with the negative controls (Figure 3A). The result showed that CCAT2 knockdown inhibited the transcriptional activity of Wnt/β-catenin signaling pathway. As proposed by previous study, β-catenin as the key transcriptional activation component of Wnt/β-catenin signal pathway was often found nuclear accumulation upon upstream activation. We next measured β-catenin expression levels in nuclear and cytosolic fractions from U87-MG and U251 cells under the manipulation of CCAT2. In coordination with previous study, as shown in Figure 3B and 3C, knockdown of CCAT2 in the stable expression cell lines with CCAT2 shRNA suppressed the β-catenin translocation from cytoplasm to nucleus, while there is little effect on the total cellular β-catenin. Subsequently, qRT-PCR and Western blot analyses showed that CCAT2 knockdown significantly decreased the levels of downstream β-catenin target genes c-Myc (Figure 3D), MMP-7 (Figure 3E) and CyclinD1 (Figure 3F) both at transcriptional and translational levels. These data indicated that the inhibitory effect of decreased expression of CCAT2 on malignant phenotype of glioma cells may be through a repression of Wnt/β-catenin signal pathway.

## DISCUSSION

Recent studies have extensively investigated the regulatory roles of CCAT2 in several types of cancer origination and progression. In the present study, we found that CCAT2 was abundantly expressed in glioma tissues and positively correlated with advanced tumor stage. In addition, loss of function assay revealed that knockdown of CCAT2 significantly inhibited glioma cell proliferation and tumorigenesis. These findings suggest that CCAT2 functions as an oncogene in glioma origination and development, and it may be a new biomarker in the glioma paradigm.

Until now, it has recently reported lncRNA functions in diverse functions, including chromatin modification, gene transcription regulation and post-transcriptional regulation of RNA splicing [13–16]. Moreover, accumulating studies have demonstrated the aberrant expression of lncRNAs in various human tumors, including colorectal cancer [17], liver cancer [18], breast cancer [19]and lung cancer [20]. For instance, lncRNA HOTAIR (Hox transcript antisense intergenic RNA) controls gene expression by interacting with a Polycomb Repressive Complex 2 (PRC2) and high levels of HOTAIR expression was investigated in multiple tumors pathogenesis and progression [21–23]. Metastasis-associated lung adenocarcinoma transcript1 (MALAT1) was firstly characterized as one of the cancer-associated lncRNAs that played crucial roles in several cancer types [24–26].

CCAT2, a novel noncoding RNA mapping to 8q24 chromosomal region, was originally detected as markedly high level in colorectal cancer. Recent evidences have showed that upregulated expression of CCAT2 contributed to various human cancers development, such as cervical cancer [27], non-small cell lung cancer [28], gastric cancer [11], esophageal squamous cell carcinoma [9, 29], etc. However, little is known about the correlation between aberrant expression of CCAT2 and biological function in glioma. In the current study, we found that CCAT2 was upregulated in glioma tissues compared to matched adjacent tissues. To further understand the biological function of CCAT2 in glioma, we performed a series of functional assays. The decreased CCAT2 expression led to the inhibition of cell proliferation, colony formation, cell cycle progression and migration. Similar efficiency of cellular growth behaviors of glioma was also observed *in vivo* that the CCAT2 knockdown decreased tumor formation.

In recent years, lncRNAs have attracted attention to understand the functional implications in various cell biology, particularly in cancer. The molecular mechanisms by which lncRNAs drive evolution, development and caners are diverse. A growing body of studies have indicated the underlying molecular mechanisms of CCAT2 in cancer origination and development by regulating the Wnt/β-catenin signaling pathway [10]. The Wnt/β-catenin signal pathway has been demonstrated to be involved in the normal embryonic development and carcinogenesis [30, 31]. Β-catenin as a key downstream effector of Wnt/β-catenin signal pathway often translocates to nucleus from the cytoplasm and involves in the regulation of carcinogenesis process by participating in cell-cell adhesion and signal transduction [32, 33]. To explore the explicit molecular mechanism of CCAT2 in glioma origination and development, LEF/TCF reporter vector was constructed and transfected into the glioma cells. CCAT2 downregulation significantly decreased the promoter activity of Wnt pathway induced by LiCl. Downstream β-catenin target genes c-Myc, MMP-7 and cyclinD1 expressions were subsequently examined. Consist with previous results; β-catenin expression in nuclei was remarkedly decreased when the CCAT2 was knocked down using shRNA construct in the glioma cells cells. Additionally, the mRNA levels and protein expression levels of c-Myc, MMP-7 and cyclinD1 were significantly decreased in stable expression glioma cells with CCAT2 shRNA. Through combining our above results, we speculated that abnormal expression of CCAT2 could inhibited the Wnt/β-catenin pathway by suppressing the downstream genes expression of Wnt/β-catenin signaling pathway in glioma cells, thus play significant roles in glioma origination and development.

In summary, the findings from our study demonstrated that the down-regulation of the CCAT2 expression inhibits cell proliferation and tumorgeniesis potential by inhibiting the Wnt signaling pathway. CCAT2 expression offers insight into a potential biomarker for diagnosis in glioma patients.

## MATERIALS AND METHODS

### Ethics statement

The approval of this study protocol was also approved by the Ethics Committee of Nanchang University and the written informed consent was obtained from all subjects enrolled in the current study. In addition, all experiments protocols were carried out in accordance with the relevant guidelines and regulations.

### Patient samples

Patients with glioma (n=134) including 94 males and 40 females who underwent initial surgery in the Second Affiliated Hospital of Nanchang University were retrospectively selected for this study. The median age of patients was 55 years ranging from 11 to 81 years. No patients had received any therapy before surgery. All tumors were classified on the basis of the WHO criteria for tumors of the central nervous system. The clinicopathological features of patients were summarized in Table 1.

### Cell culture

The human glioma cell lines (U87-MG and U251) were purchased from the Cell Bank of Chinese Academy of Sciences (Shanghai, China) and cultured in the DMEM medium containing 10% fetal bovine serum in an incubator at 37°C and 5% CO_2_.

### Isolation of cytoplasmic and nuclear RNA

Cytoplasmic and nuclear RNA from glioma cell lines U87-MG and U251 were separated and purified using the Nuclear/Cytosol Fractionation kit (Biovision) according to the manufacturers' instructions. The primers used for quantifying CCAT2 expression were 5’-CTTCCAGCTCCACCTCTGAC-3’ (Forward) and 5’-GAGCTCAAAGGACGATGAGG-3’ (Reverse).

### RNA extraction and quantitative real-time PCR (qRT-PCR)

Total RNA was extracted using TRIzol reagent (Invitrogen, Carlsbad, CA) following the manufacturer's instructions. The ratio of absorbance at 260 nm to 280 nm (A260/A280 ratio) of the isolated RNA measured all over 2.0 using NanoDrop ND-2000 spectrophotometer was used to synthesize the first-strand cDNA by M-MLV reverse transcriptase (Invitrogen). About 1 ug of total RNA in a final volume of 20ul reaction mixture was reverse transcribed. After reverse transcription, quantitative real-time PCR was performed to quantify the expression levels using SYBR Premix ExTaq II (TaKaRa). The relative gene expression from each example was calculated using the 2^ΔΔ CT^ method from three independent experiments. Glyceraldehyde-3-phosphate dehydrogenase (GAPDH) was used as an internal controls.

### Western blotting

The total cellular, cytoplasm, and nuclear proteins were prepared from glioma cell lysates with lysis buffer and subjected to 10% SDS-polyacrylamide gel electrophoresis (SDS-PAGE) gel. Western blot analyses were performed with the following primary antibodies polyclonal antibodies against β-catenin (1:2000 dilution), c-Myc (1:3000 dilution), MMP-7 (1:1000 dilution) and CyclinD1 (1:2000 dilution) from Santa Cruz Biotechnology, USA as previously described.

### Plasmids, shRNA and transfection

To obtain the shCCAT2-expressing cells, the pGV248-CCAT2 shRNA and scramble shRNA obtained by Genepharma (Shanghai, China) were respectively transfected into 293T cells along with the packaging plasmids. The lentivirus partials were harvested and the knockdown efficiency was determined by qRT-PCR after co-transfection 48h. The lentivirus with pGV248-CASC2 shRNA or scramble shRNA was then used to infect glioma cells to construct stable expression cell lines for functional analysis.

### Cell proliferation assay and colony formation assay

The stable expression cell lines with pGV248-CCAT2 shRNA or its respective controls (2,000/well) were grown in 96-well plates. Cell viability was determined by Cell Counting Kit 8 (CCK-8, Dojindo, Japan) according to the manufacturer's instructions at indicated time points (1, 2, 3 and 4 days), respectively. For the colony formation assay, 200 stable expression cells as described above were plated on 6-well plate and maintained in medium containing 10% FBS for two weeks. More than 50 cells were defined as a positive colony and the number of clones was counted in separate wells in triplicate.

### Flow cytometric analysis

The stable expression cell lines with CCAT2 shRNA or its respective controls were assessed for cell cycle analysis by using a flow cytometer (BD Biosciences) after cells were stained with Propidium iodide (PI). Every experiment was repeated three times.

### Transwell assay

Cell migration was determined by the transwell assays using transwell filters (BD Biosciences, San Jose, USA). A sample of 3×10^4^ cells suspended in a serum-free DMEM was transferred on the upper well and DMEM containing 20% fetal bovine serum was added to the lower well as chemo-attractant. After 48h, the migrated cells on the lower surface were fixed with methanol, stained with 0.4% crystal violet and counted. Three independent experiments were analyzed.

### *In vivo* assay

Twenty-four nude mice were purchased from Chinese Academy of Science Shanghai Experimental Animal Center. Experimental animals were fed under standard guidelines at the local and national regulations. Mice were randomized divided into four groups. Glioma cells (1×10^7^ in 100ul medium) stably expressing CASC2 shRNA or control vector were subcutaneously injected into the BALB/C female nude mice with 6 weeks old. Tumor volume in each mice was measured every 3 days by taking two of its dimensions (tumor volume=length×width×width/2).

### Statistical analysis

Statistical analysis was performed using the SPSS Graduate Pack, version 11.0, statistical software (SPSS). Two-tailed paired Student's t test and one-way ANOVA were used for analyzing for comparison different groups. Data are expressed as means±standard deviation (SD) of three independent experiments. *P* values less than 0.05 was considered to be significant.

## SUPPLEMENTARY MATERIALS FIGURE


