# Supplementary material for: Knockdown of long non-coding RNA CCAT2 suppressed proliferation and migration of glioma cells
Source: Oncotarget. 2016 Nov 9;7(49):81806–14. doi: 10.18632/oncotarget.13242 (PMC5348431; doi:10.18632/oncotarget.13242)
Supplement: Supplementary file 1 [file oncotarget-07-81806-s001.pdf]

Knockdown of long non-coding RNA CCAT2 suppressed proliferation and migration of glioma cells

SUPPLEMENTARY FIGURE

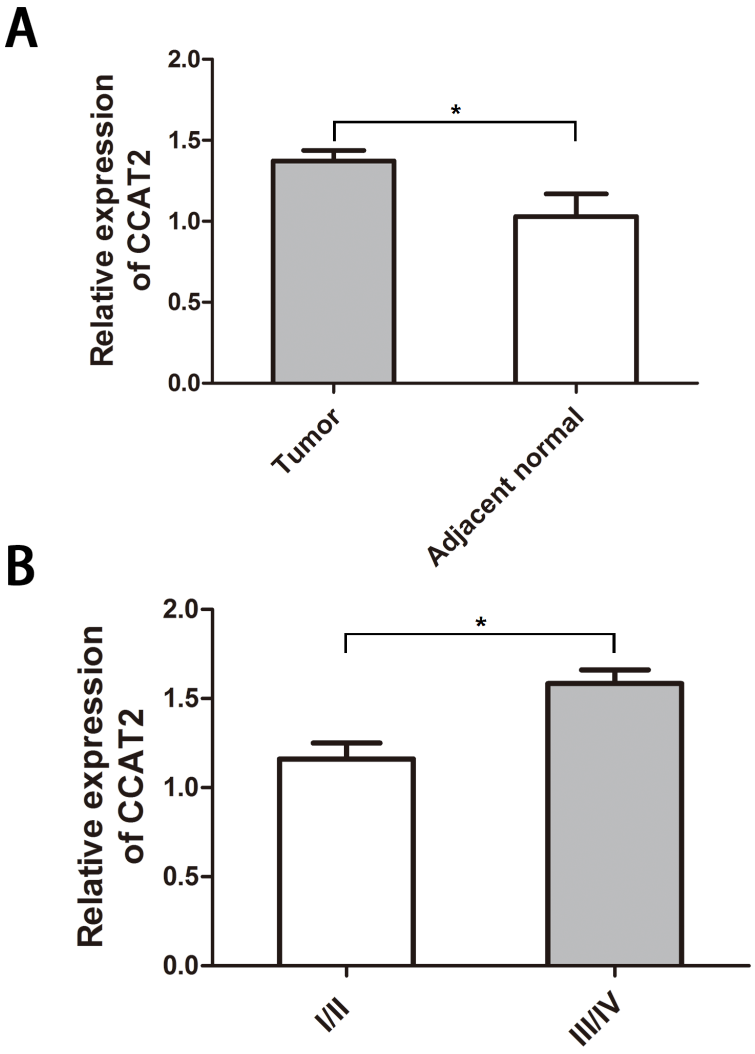

Supplementary Figure S1: The expression levels of CCAT2 was analyzed by qRT-PCR in 56 paired glioma tissue samples and patients with advanced TNM stage was correlated with increased CCAT2 expression.
